# The ‘Murky’ New Orleans: A community reliving and experiencing the 2005 Hurricane Katrina

**DOI:** 10.4102/jamba.v14i1.1396

**Published:** 2022-11-25

**Authors:** Alice Ncube

**Affiliations:** 1 Disaster Risk Management Training and Education Centre for Africa, Faculty of Natural and Agricultural Sciences, University of the Free State, Bloemfontein, South Africa

This captivating book makes good reading on the disaster (risk) management field, and the authors, Kai Erikson, the William R. Kenan, Jr. Professor Emeritus of Sociology and American Studies at Yale University, and Lori Peek, a professor of sociology and the director of the Natural Hazards Center at the University of Colorado, are best positioned to interrogate the subject. *The continuing storm: Learning from Katrina* is applicable in the current period where naturally induced and man-made disasters are on the increase globally.

As the title suggests, the continuing storm will linger on in the lives of the people of New Orleans, the United States of America (USA) and the world because of its impact on the body, mind and soul. This is a reminder of how human activities place us in harm’s way in terms of recurrence, exposure and hazard frequency. As part of the Katrina bookshelf series edited by Kai Erikson, this captivating and engaging book by Kai Erikson and Lori Peek will make the reader think more about the human race as defined by the biblical aspects of humanity and how human beings define a society. Human beings are all the same, but in this book, they are defined by race and colour. The socio-economic stratification of life is blatantly manifesting in a developed country.

Hurricane Katrina revealed many human challenges that preceded the hurricane’s landfall. These are systemic racism, poverty and gender inequality; corporate greed and political corruption; residential segregation and collective abandonment of public institutions; resource extraction and unchecked environmental degradation. Katrina did not cause these and so many other social ills. They were the precursors to the immense suffering that followed in the storm’s wake.

The stimulating piece of work contained in the book titled *The Continuing Storm: Learning from Katrina* by Kai Erikson and Lori Peek can be valuable to students, lecturers, practitioners and policymakers in the disaster management field. The three-part book (part 1: A hurricane known as Katrina; part 2: Locating Katrina; and part 3: Katrina as human experience) brings to the fore the intersection of nature and humanity, as theoretically represented in the traditional disaster management continuum and the pressure and release (PAR) model (1994). The vivid description of the physical, mental and emotional impacts caused by Hurricane Katrina brings to the fore the new thinking needed in the 21st century, whereby climate change discussions are no longer farfetched but realities. The book also highlights how climate change has become rhetoric and is no longer an issue of deep science and technology alone but also a social issue. It is also interesting that this novel compilation is set in a country considered a pioneer in world development. For students, especially in disaster management, the book will assist in revealing the causal factors of disasters. The naturalness of disasters and the debate around the human influence on disasters are discussed. This is a reminder that the disaster phenomenon is because of human involvement and interference. The book is ideal in the study of disasters and the influence of man on how disasters unfold. It is no coincidence that the authorities made the hurricanes’ path easier. The authors reiterated this by saying:

Human engineering has often turned to straight lines where curves seem to have been nature’s own way and that certainly appears to have been the case in New Orleans and across the Louisiana wetlands. (p. 51)

Those interested in environmental and ecological studies can also benefit from the book. The community participation aspect came up in Chapter 2, which showed that the people of New Orleans assisted each other without outside help, indicating a resilience in communities that is undermined. This challenges some definitions of a disaster that state that hazards escalate into disasters if the affected communities cannot cope with their resources; in this instance, the book can enhance discussions on the issue of strengthening community coping capacities and enhancing community resilience.

A disaster like Hurricane Katrina can tell us a great deal about the basic core of the American way of life. The interdisciplinarity of the research space nowadays makes the book more relevant than ever. The book clearly interrogates the intersectionality of nature, the human being and the development trajectory that could possibly be the reason for disasters of this magnitude. Hurricane Katrina changed the landscape of New Orleans forever as it pounded the city and the surrounding area. The book highlights the way the community of New Orleans is viewed by the broader American society and the world at large. After the hurricane, their lives have never been the same. The dislocation of the New Orleanians was both physical and emotional.

The book presents the events of the hurricane in interesting and captivating nuance. The disaster is considered ‘… as if the entire Gulf Coast were obliterated by the worst kind of weapon you can imagine’ (former US president George W. Bush (p. 3)); ‘… place is going to look like little Somalia’ (*Army Times* report (p. 34)); ‘… like Baghdad on a Bad Day’ and ‘the urban insurgency the US military currently [*at that time*] faces in Iraq’ (*Washington Post,* p. 35). One government official, Lt. Col. John Edwards of the Arkansas National Guard, said of the disaster, ‘It reminded me of the liberation of France in World War II’ (p. 37). The book is also recommended for the developing south region, particularly in sub-Saharan Africa and South Africa, where the book is timely considering the recent disasters. Cyclones Idai and Kenneth affected Mozambique, Zimbabwe and Malawi in 2019 and 2022; floods affected KwaZulu-Natal and the Eastern Cape in South Africa.

Media and communication students and practitioners can also benefit from the book. There is clear evidence of how information can be distorted, which could result in more fatalities and damages than if accurate information had been made available. The media from outside the disaster area sensationalised the whole issue, and sadly, the government bought it. The media has the primary role of informing the public of what is happening so that a correct response can be undertaken. There were reports of massive looting, rape cases and vandalism of infrastructure. The reality was that the people of New Orleans needed life-saving items like water, food and other necessities to keep their families and themselves alive, items described by one as efforts to means of subsistence. According to one account, the most widely ‘looted’ items were disposable diapers, dry shoes, clothing, foods of all kinds and medications. The people did not focus on looting guns, jewellery or television sets. ‘To meet basic needs’ (p. 22) is undoubtedly the best answer to why people did what they did. Misinformation led to slowing down troop deployments to New Orleans as well as medical evacuation.

Such distortion of information highlights the deep-rooted inequalities that characterise the USA. This also became known when relief efforts were rolled out. The response and relief efforts that were undertaken by the locals were termed looting and stealing, while outside assistance was termed commandeering. This was articulated by Kanye West, an often outspoken rapper, who said of the media during a live television broadcast: ‘If you see a Black family, it says they’re looting. If you see a White family, it says they’re looking for food’ (p. 24). A church deacon named Harold Toussaint said in an interview: ‘Yeah, it was interesting. If you were White, you were commandeering a boat. If you were Black, you were stealing it’ (p. 24). This highlighted the way black Americans are dehumanised and called all sorts of names.

The book highlights the great divide in society, which is according to colour lines, that is, black and white. Black people are seen as ‘looting’ food while white people ‘find’ food. All this information was unfounded and proven to be untrue. The authors highlighted that disaster has a way of bringing out the best and the worst instincts in the news media. This also reminds us that misinformation can be costly to individuals through loss of lives, the community through loss of belongings and the nation through misdirecting assistance.

The book highlights racial issues in the country as survivors were termed refugees, which is contrary to the official refugee definition. The residents of New Orleans were citizens of America who were displaced by a disaster. However, the way it was portrayed indicates that black residents of New Orleans were taken as outsiders and that their lives mattered less, a suspicion that was raised all along. This was because services were offered based on racial lines. The book highlights the figurative language that marred this period of the disaster. The authors highlight the *lingua* [*tongue*] that characterised the response and relief period in Hurricane Katrina, that is: *Them; Other; They; You Know Them*, to simply denote the black Americans affected by Hurricane Katrina.

There is adequate evidence presented in the book that it is no longer business as usual. It would be an understatement to say that climate change is real, and with this book, the issue of sustainable living as an urgent agenda item for policymakers comes to the fore. As the title says, the continuing storm will linger in the lives of the people of New Orleans, the USA and the world over because of the impact it had on the body, mind and soul. This captivating book will make the reader think more about humans as defined by the biblical aspect that humanity is one. This book brings to the fore the biblical definition of human beings as all are created in the image of God.

The book is an easy read as both a hard copy and soft copy. The language is very easy to understand, and the authors clearly articulate scenes and other events that preceded, took part in and extended the disaster occurrence. The style is suitable for tertiary students going forward, and as plans are in place for the mainstreaming of disaster management into the South African secondary school curriculum, this book can be useful for high school learners. The setting of the book is very captivating and fit for this purpose.

The first chapter offers an overview of what happened as the disaster was unravelling, ‘Along the Shores of the Gulf’ (Chapter 1), which started in the early morning of 29 August 2005, along the coasts of Louisiana, Mississippi and Alabama. Katrina wiped out 36 square miles of Louisiana wetlands, which for millennia had been nature’s way of protecting the terrains further inland against disturbances coming from the open sea. Katrina was also responsible for 10 oil spills, which collectively spewed a volume of petroleum into the environment larger than other spills on record in the United States (more than two-thirds of the amount poured out in the Exxon Valdez oil spill of 1989).

The second chapter is much longer than the first, because in many ways, the story of New Orleans became the story of Katrina, no matter how out of balance that may feel at first. For one thing, New Orleans was by many measures the worst casualty of that disaster in both the short term and the long term. For another, the suffering that was on full display on the streets of New Orleans became a national and even an international symbol of urban poverty and racial injustice.

Chapter 3 asks when a disaster like Katrina started and when it ended. How should it be located in time? Commentators generally mark the pastness of disasters like Katrina by noting their anniversaries, and almost every account of the disaster known as Katrina – including the one we offer here in this prelude – opens with the fateful date of 29 August 2005. Thus, August 2015 was the 10th anniversary of Katrina; August 2025 will be the 20th, and so on.

Chapter 4 relates the events as they take place in space, that is, the geographical space of New Orleans and the living organism New Orleans. Chapter 5 relates how the rule of the jungle was applicable to the people in the eye of the storm:

The storm came directly to me, flood water were pursuing them like a living creature; water was like a demon, winds and water came to the city, and the storm was out to get them. (pp. 73–74)

As described by one resident:

That water didn’t knock or ring the bell. It chased me up the stairs, into the attic, and onto the roof. Water is not supposed to act like that! I could have sworn it was mad at me. (p. 74)

There is social stratification in this community, and this is according to the colour of the skin. It is said to be a stable social pattern – where the poor are located. Furthermore, the general rule that applies in disasters and the development realm has an exception that is indicated in New Orleans, that is, the well-off are known to deliberately place themselves in harm’s way when they can afford the risk and there are benefits to be gained. Disasters may deepen already existing inequalities, pushing struggling people even further into the depths of economic despair.

Chapter 6 gives an account of how the storm battered the people who were overpowered by nature. The damage to the community includes not only damages to the body but also the mind. One account states, ‘I could stand on my back porch and scream at the top of my lungs and not hear myself’ (p. 84). Finally, Chapter 7, the final chapter of the book, gives an account of the pains of being displaced in your habitat, that is, natural location, by the mere fact that this was the only earthly home the residents knew. The poorest segments of the population were living with the greatest risk and were the most likely to have to confront the storm head-on. Some sentiments portrayed a sense of suffocation that can result from social isolation. James, one of the evacuees, felt ‘trapped and alone’ (p. 101) in what was a wholly unfamiliar location: ‘The work of the public sector to provide needed relief and assistance “was” itself a source of profound disorder and confusion, a kind of a second disaster’ (p.102). In the end, they raise a critical query: ‘How does one recover from “recovery”?’ (p. 102).

As part of the disaster management discourse, it could have been interesting to get perceptions and accounts of the authorities in power 15 years after the disaster happened. Many other disasters similar to the one accounted in the book and others like wildfires, migration challenges and coronavirus disease 2019 (COVID-19) have occurred in the same country. The accounts of these other disasters seem to be indications of what happened when the warnings about the Hurricane Katrina was issued. A chapter, perhaps, on the Federal Emergency Management Services (FEMA) and other notable authorities could have formed perhaps the last and final chapter of the book. Furthermore, they authors could have extended the book a bit and given a well-thought-out section of the Ecosystem-Based Disaster Risk Reduction Climate Change Adaptation and Agenda 2030. Not taking away anything from the well-thought-out book, the authors could consider looking at terminology like rehabilitation, reconstruction, development and redefining the development agenda in the Fourth Industrial Revolution (4IR). Finally, the question of what now to those who would never return to New Orleans could be addressed by sociologists and anthropologists.

The book can make a major contribution to policymakers at local, regional and international levels. This is because the authors tried to show the early warning systems (EWSs), effective response mechanisms, coping and adaptation capacities and building better principles that can be incorporated in disaster (risk) management planning. The chaos and confusion caused by the media highlights how the principles of proper impact assessment are to be emphasised rather than unorthodox ways of uninformed response and relief processes that resulted in increased fatalities and worsened destruction because of the hurricane.

Although the book is set in the USA, it portrays inequalities that are unthinkable in a developed country and happening in the 21st century; the book is a valuable tool that can benefit avid readers and analysts of disaster management discourse, philosophers, sociologists and academics in various fields like engineering. Policy implementers and engineering are mentioned specifically because they are among the implementers and scientists who decided not to heed the advice to not develop the area artificially by using man-made levees and other barriers to block the storms from reaching the communities in New Orleans. Generous mention of the live reports of the people who were displaced by the disaster is useful to students and educators in disaster management. For undergraduate students, the book can be useful for learning the basic concepts of hazards and disasters. For postgraduate students, the book lends itself to expand critical analyses of development and sustainable living in the 21st century considering that climate change and adaptation discourse is in every agenda of living.

Finally, for disaster management students at both the tertiary and high school levels, the book is a good reference point to learn and critique decision-making in theory and in practice. As for the practitioners and policymakers, the book will remind them of the basics in terms of planning and implementation of projects. The sociologists and anthropologists can make reference to this book as they reminiscence about the journey towards sustainable living for all in the 21st century in pursuance of the sustainable development goals.

